# Shaking table tests of a one-quarter scale model of concrete hollow block masonry houses retrofitted with fiber-reinforced paint

**DOI:** 10.1038/s41598-024-58365-4

**Published:** 2024-04-05

**Authors:** Zamzam Multazam, Kenjiro Yamamoto, Kishor Timsina, Chaitanya Krishna Gadagamma, Kimiro Meguro

**Affiliations:** 1 Institute of Industrial Science, The University of Tokyo, Tokyo, Japan; 2 Department of Civil Engineering, The University of Tokyo, Tokyo, Japan

**Keywords:** Civil engineering, Natural hazards

## Abstract

Unreinforced masonry (URM) buildings are prone to significant damage when subjected to ground motion. Some strengthening methods have been proposed to increase the seismic capacity. However, the widespread adoption of these methods faces various challenges, including economic constraints experienced by common people in developing countries, the complexity of implementation, efficiency, and seismic safety of each technique. This paper introduces a new retrofitting method of fiber-reinforced paint using fiberglass as the primary reinforcing material. The advantage of this technique lies in its simplicity and ease of application, with the added benefit of using the paint to improve the appearance of the house. Two 1:4 scale concrete hollow block (CHB) masonry houses were constructed to represent unreinforced masonry and retrofitted masonry structures using fiber-reinforced paint (FR-Paint). The shaking table test results indicate that the retrofitted house model showed improvements of up to 18 times in deformation capacity and up to 13 times in energy dissipation compared to the non-retrofitted house model. FR-Paint has a robust performance even in high input motion at a seismic intensity JMA of 7 (Japan Meteorological Agency). This confirms that this retrofitting method has a high earthquake-resistant performance.

## Introduction

Numerous studies have been conducted to investigate the collapse of masonry structures during earthquakes and to develop mitigation strategies, leading to the establishment of new building codes^1^. Nevertheless, a substantial number of masonry structures were constructed before the new standards, and in many cases, they do not adhere to the established building codes— a common practice, particularly with untrained labor in developing countries^1^. This type of construction lacks engineering intervention and is typically referred to as non-engineered building. The building collapse results in a huge number of human casualties during earthquakes, with unreinforced masonry structures making a significant contribution to this statistic^2,3^.

The current study focuses on concrete hollow block (CHB) masonry structures, commonly found in residential houses in developing countries^4^. However, CHBs exhibited limited compressive strength; for example, in the Philippines, CHBs have a weak compressive property, measuring 1 MPa^5^. Common failure behavior in masonry houses is out-of-plane wall failure, in-plane shear failure, and a combination of in-plane or out-of-plane failure of walls, and roof collapse^6,7^. A shaking table test was carried out on unreinforced masonry houses made of CHB, revealing that the gable wall was the weak element, ultimately leading to its collapse during the shaking^5^. Despite the building being reinforced, masonry walls often experienced heavy damage due to the low compressive strength of CHB. An illustrative example is observed in a school building (reinforced concrete frame), where no significant damages were incurred by the frame, but the masonry units were broken and fell, posing a significant risk to life^4^.

Improving the seismic resilience of unreinforced masonry structures is a critical priority for earthquake disaster mitigation. Several factors must be taken into account when selecting a retrofitting method, including enhancing the seismic capacity of structures, ensuring affordability for the common people in developing countries, local availability of materials, feasibility for unskilled labor, and cultural acceptability^8^. Many studies have investigated the performance of various retrofitting techniques for URM structures, including shotcrete reinforcement, grout/epoxy injection, post-tensioning using rubber tires, and confinement. While these methods have their advantages and disadvantages, many of them present challenges during implementation.

Masonry walls constructed with half-scaled single wythe hollow clay brick, retrofitted by shotcrete, were tested under in-plane loading. The findings show that shotcrete can improve the deformation capacity, ultimate lateral strength, and energy dissipation of masonry walls. However, the process of shotcrete retrofitting is time-consuming and causes significant disturbance to surrounding areas^9^. Grout or epoxy injection is used to repair damaged masonry. This approach has been proven to restore the strength of masonry houses. However, the injection process, which involves applying pressure, remains a challenging aspect for individuals without specialized skills or training, particularly in developing countries^10,11^. Precast reinforced concrete (RC) has been used to improve the seismic capacity of URM structures by enclosing them with precast RC elements^12^. This approach can improve the deformability and energy dissipation of URM. The placement of precast RC and their spacing significantly influence the behavior of URM^12^. This technique is appropriate for new masonry constructions. However, for existing buildings, it involves demolishing and rebuilding the walls, which makes it less practical for retrofitting. Polypropylene band (PP-band), commonly used for packing, was arranged in a mesh and embedded in a cement mortar overlay. Two different full-scaled masonry walls were constructed and subjected to shear wall testing^13^. 1/4 scaled models of masonry houses of non-retrofitted and retrofitted using PP-band were evaluated by shaking table test^14^. PP-band can increase the deformation capacity and energy dissipation of URM. Although PP-band is cost-effective technique, the construction process demands time and skilled labor. A study involved testing 1/10 scaled URM houses retrofitted with rubber straps in both horizontal and vertical directions, applying a stretching force of 50 N by hand^15^. This retrofitting method effectively enhances the deformation capacity of unreinforced masonry (URM), making it more ductile with gradual damage formation^15^. Despite the affordability and availability of rubber straps, challenges emerge in ensuring the precise and controlled application of force to achieve the desired level of stretching, without causing damage or compromising the effectiveness of the retrofitting technique. Additionally, concerns related to fire safety and the appearance of masonry houses remain issues in this retrofitting approach.

Many studies have explored the effectiveness of Fiber Reinforced Polymer (FRP) in the form of strips or fully covering walls for unreinforced masonry. The extent of improvement depends on the ratio of FRP, the cost rises with the greater quantity of FRP used. Glass Fiber Reinforced Polymer (GFRP) and Carbon Fiber Reinforced Polymer (CFRP) as retrofitting materials for masonry walls have been investigated, utilizing various techniques including full surface, strips, and near surface-mounted installations (NSM)^16–20^. The full surface application of GFRP and CFRP fabrics exhibited a greater improvement in shear strength compared to strips cut from fabrics^17,20^. A typical failure mode of this method involves debonding between the FRP layer and the wall surface. Following this debonding, the FRP is no longer attached to the wall, thereby initiating masonry failures. Brittle failure occurs when the specimen reaches its ultimate strength. The bond behavior of FRP in clay brick masonry has been investigated, utilizing GFRP strips for externally bonded and CFRP strips for NSM tests^21^. This selection was made based on material availability and ease of preparation. The results indicate that the NSM retrofitting technique is considered effective for improving bond behavior. However, the installation required extensive and careful preparation, involving wall surface conditioning, embedding the strips within masonry, and aesthetic consideration^21^. Additionally, FRP was used for repairing and retrofitting damaged unreinforced masonry. In-plane testing was conducted to compare original unreinforced masonry (URM), retrofitted specimens, and repaired damaged URM using FRP strips. Despite the repairs, cracks still developed in the unrepaired parts of the masonry^22,23^. The combination of PP-band and FRP strips as retrofitting materials has been proven effective in enhancing the deformation capacity, energy dissipation, and ductility of structures. However, a greater quantity of FRP is required to further improve the seismic capacity of URM^24^.

Alternative retrofitting materials involve mixing cementitious material with fibers, such as Engineered Cementitious Composites (ECC), Fabric-Reinforced Cementitious Matrix (FRCM), and Textile-Reinforced Mortar (TRM)^25–29^. Fiber is an effective additive for enhancing the strength of cementitious materials. Cementitious materials are strong under compression but are vulnerable to tension. When combined with fibers, the fibers bear the tensile load, preventing the crack propagation. The effectiveness of the bond between the cementitious material and fibers is crucial in ensuring the strength of the resulting composite^30^. It demands highly skilled labor to achieve optimal results.

This study considers several factors when determining retrofitting materials. Fully covering the entire masonry surface with retrofitting materials is crucial to prevent crack propagation. The retrofitting material must possess a strong adhesive quality capable of securing the connection between the retrofitting materials and the masonry surfaces. Additionally, the technique should be easy to implement, ensuring practical and feasible for real-world applications in developing countries. This study introduces a novel retrofitting approach involving the combination of fiberglass, paint, and resin, referred to as fiber-reinforced paint (FR-Paint). Considering painting as a common practice in construction to enhance the appearance of a house, the incorporation of fiberglass into the paint has the potential to foster the widespread adoption of this technique. The current study discusses the shaking table test of two 1/4 scaled models of one-story CHB masonry houses with and without retrofitting by fiber-reinforced paint. Using a fiberglass ratio of 1% and a coating thickness of approximately 1 mm, the experiment aims to provide insights into the behavior of one-story URM and FR-Paint retrofitted masonry houses when subjected to dynamic loading.

## Experimental plan

### Scale factors

The shaking table at the Institute of Industrial Science—the University of Tokyo features dimensions of 1.5 × 1.5 m and can support a maximum mass of 2000 kg. Given the limitations preventing a complete full-scale analysis, this experiment was conducted at a reduced scale of 1:4. In a scaled modeling experiment, two commonly used similitude criteria are the Cauchy and Froude similitude laws. Cauchy laws consider the relationship between inertial and elastic restoring forces, whereas Froude's law considers the relationship between inertial and gravity forces^31^. Both criteria are desirable for experimental studies. However, using both Cauchy-Froude similitude laws requires additional masses, that exceed the capacity of the available shaking table. Therefore, for the current study, it was decided to utilize the Cauchy similitude law, as it has been widely used in similar experiments^14,24,32–34^.

**Figure 1:**
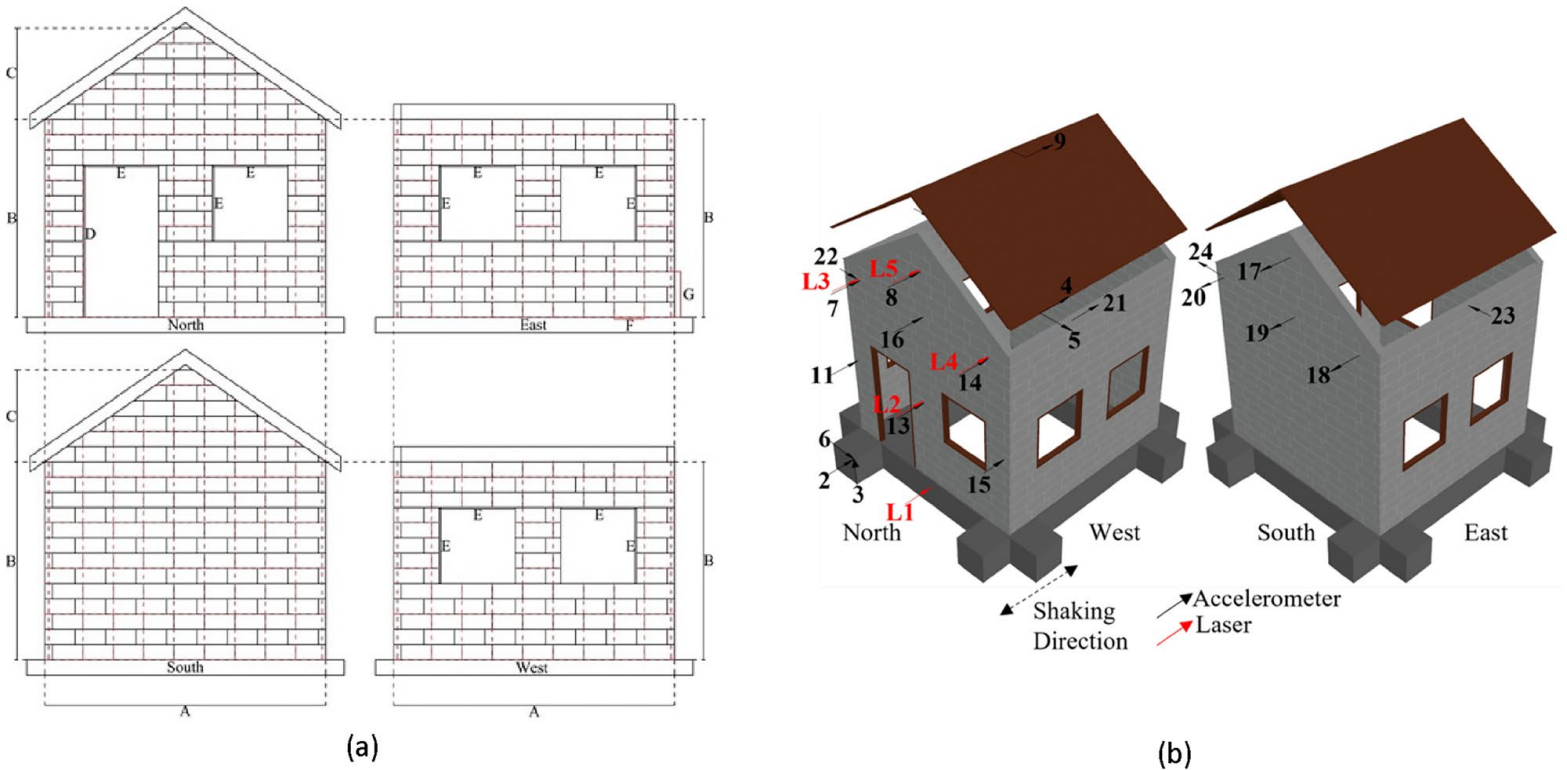
(**a**) The geometry of the house model: A, B, C, D, and E represent the floor dimension (0.9 m), the story height (0.65 m), the gable height (0.3 m), door dimension (0.5 m), and window dimension (0.25 m); (**b**) position of the accelerometers and lasers for the specimens.

The scale factor was calculated by dividing the parameter in the prototype ($p$) by the corresponding parameter in the model ($m$). In designing the models, the fundamental requirements for achieving similar dynamic responses and failure behavior involve ensuring similarity in the distribution of mass and stiffness between the prototype and the model. In a scaled-down experiment, introducing additional mass is crucial to meet the criteria of similarity of mass distribution^35^. However, the incorporation of a significant additional mass in the experiment is not possible due to the restricted mass capacity of the shaking table test. Consequently, it becomes crucial to consider material scaling, achieved through modifications in material strength. Without appropriate material scaling, the scaled model may become stronger, demanding heavier loads to induce failure. The stronger model can result in stress distributions that do not accurately represent prototype, different failure mechanisms, and unexpected patterns of damage^36^. Therefore, to replicate the failure behavior of the prototype, the strength of the model was reduced following the scale factor (${S}_{f}={S}_{L}=4$), which has been employed in previous studies^14,24,37^. The low-strength characteristics of CHB can result in the failure of individual masonry units. In this study, to replicate the same failure pattern, compressive strength of both CHB and mortar was deliberately reduced. Considering micro-concrete as the selected material, the scale factor of density is equal to unity (${S}_{\rho }=1$)^38^. The acceleration scale factor is equal to unity (${S}_{A}=1$) since the gravity in both the prototype and the model remains the same (${g}_{p}={g}_{m}$). The scale factor calculations for other quantities are summarized in Table 1. A simple house model was constructed, resembling typical residential homes in the Philippines^5^, as illustrated in Fig. 1. The actual size of CHB is 400 mm × 200 mm × 100 mm, and the model dimensions were scaled down to 100 mm × 50 mm × 25 mm based on the scale factor. The geometric and mechanical properties of the prototype and model are given in Table 2.

**Table 1:** Modeling scale factors.

Physical quantity	Modeling factor		
	Relationship	True model	Current study
Length $(L)$	${S}_{L}={L}_{P}/{L}_{M}$	4	4
Specific mass $(\rho )$	${S}_{\rho }={\rho }_{P}/{\rho }_{M}$	1	1.04
Force $(F)$	${S}_{F}{=S}_{L}^{2}{S}_{f}$	64	57.6
Displacement $(d)$	${S}_{d}={S}_{L}$	4	4
Strain $(\varepsilon )$	${S}_{\varepsilon }={\varepsilon }_{P}/{\varepsilon }_{M}$	1	1
Strength $(f)$	${S}_{f}={f}_{P}/{f}_{M}$	4	3.6
Acceleration $(a)$	${S}_{a}={S}_{f}/({S}_{L}{S}_{\rho })$	1	0.87
Velocity $(v)$	${S}_{v}={({S}_{\varepsilon }{S}_{f}/{S}_{\rho })}^{0.5}$	2	1.86
Frequency $(\omega )$	${S}_{\omega }=1/{S}_{t}$	0.5	0.47
Time $(t)$	${S}_{t}={S}_{L}{({S}_{\varepsilon }{S}_{\rho }/{S}_{f})}^{0.5}$	2	2.15

**Table 2:** Geometric and mechanical properties of the prototype and model.

	Model	Prototype	Scale factor
Geometry			
Story height (m)	0.65	2.6	4
Gable height (m)	0.3	1.2	4
Floor area (m)	0.9 $\times$ 0.9	3.6 $\times$ 3.6	4
Wall thickness (m)	0.025	0.1	4
Concrete hollow block (CHB)			
Length mm)	100	400	4
Height (mm)	50	200	4
Depth (mm)	25	100	4
The thickness of mortar (mm)	5	20	4
Density of CHB (g/cm^3^)	1.54	1.6	1.04
Compressive strength (MPa)	0.28	1	3.6
Compressive strength of mortar (MPa)	1.94	7.92	4.09

### Materials

#### Masonry unit and mortar

The masonry units were manufactured at the Institute of Industrial Science—the University of Tokyo, intentionally reducing the compressive strength according to the scale factor by adjusting the cement-sand ratio. Utilize 3D printing to manufacture molds, as illustrated in Supplementary Fig. S1a–c online, which were then employed for the production of concrete hollow blocks. Sand No. 7 with a grain size of 0.07–0.21 mm, readily available in Japan, was used in production alongside ordinary Portland cement. These specifications were employed for both CHB and mortar production. In CHB production, to attain the desired strength of CHB, various sand and cement mixes were utilized, ranging from a cement-sand ratio of 1/7 to 1/12, with a 24-h curing time. The compression test followed the guidelines outlined in ASTM C140 standards^39^, specifically designed for Sampling and Testing Concrete Masonry Units and Related Units. The testing setup is illustrated in Supplementary Fig. S1d online. Three samples were tested for each mix category using the AG-100 KN Autograph Shimadzu testing machine. The CHB with a cement-sand ratio of 1:12 exhibited the closest strength to the target. The mortar mix, consisting of cement, sand, and water with a cement-sand ratio of 1:4 and water content adjusted to achieve optimal workability, was also employed in the previous study^5^. The cement-sand ratio was modified to 1:6 to deliberately reduce the strength of the mortar, aligning with the material scaling requirements. A cement-sand ratio of 1:4 was used as the reference strength value, while a ratio of 1:6 was implemented in this scaled-down model experiment. The compressive strength testing of mortar was conducted following the ASTM standard (ASTM C109/C109M -20)^40^, as illustrated in Supplementary Fig. S1e online. Table 2 shows the result from the compressive strength testing of CHB and mortar.

#### Fiber-reinforced paint (FR-paint)

FR-Paint was locally produced in the laboratory using materials readily available in Japan. There are two types of paint available in Japan—ordinary paint and paint containing resin. Ordinary paint is widely used for various applications including improving the appearance of building surfaces, whereas paint containing resin is typically used for specific purposes that demand enhanced durability and protection from harmful environmental conditions (e.g. corrosion, ultraviolet radiation, waterproofing, etc.). In the current study, a variant of paint containing resin was used and mixed with 1% (by weight) of 12 mm length fiberglass. The properties of the FR-Paint can be modified by varying the fiber ratio in the paint. Ordinary paint is also can be used to create FR-Paint, by separately adding resin and fiberglass. The mixing process takes at least one hour, depending on the mixing rate, and requires continued stirring until uniform consistency is achieved, as depicted in Supplementary Fig. S2b online. Supplementary Fig. S3 online shows the axial tensile testing of FR-Paint conducted following ASTM standard (ASTM D638)^41^, resulting in a measured tensile strength of 1.78 MPa for the FR-Paint. Furthermore, in-plane diagonal compression tests were conducted on masonry wallets^42^. The results revealed the significant deformation capacity of FR-Paint.

### Construction of house model and retrofitting procedure

Two identical house models, non-retrofitted and retrofitted, were constructed using the same masonry units, mortar, workmanship, and a curing period of 14 days. The dimensions and specifications of both house models are illustrated in Fig. 1a. The house model featured four walls, with gable walls on the north and south sides, and was constructed without RC frame. The north wall included a door and a window, while both the east and west walls had two windows each. Each wall was constructed with 13 layers of 9 CHBs, and the gable walls comprised 6 layers (Fig. 1a). The mortar, which has higher compressive strength than CHB (with CHB compressive strength approximately 1 MPa), was also poured into the CHB holes without compaction. It is important to note that compaction of mortar plays a significant role in determining the strength of walls^5^.

The total thickness of the FR-Paint was 1 mm with a curing period of 14 days. The painting procedure was carried out in two stages. Initially, a 0.5 mm layer was applied to the wall with a curing period of seven days, followed by an additional 0.5 mm layer applied under the same curing days. All openings, including windows and doors, were painted and connected from the outside to the inside wall. The painting process of FR-Paint demonstrated a speed and simplicity, as illustrated in Supplementary Fig. S2a–d online.

### Testing procedure

The shaking table test at the Institute of the Industrial Science, University of Tokyo, can operate with six degrees of freedom, offering frequencies ranging from 0.1 to 50 Hz and amplitudes from 0.05 to 1.4 g. Moreover, it had a maximum displacement of ± 100 mm, and the maximum mass of the specimens was 2000 kg. For simplicity, this experiment limited the shaking table test to a single direction from north to south. Twenty one-dimensional accelerometers were installed in the masonry walls of both house models, accompanied by an additional five displacement lasers positioned used off-model. Figure 1b shows the locations of the accelerometers and lasers. The data were recorded continuously during the tests, with the sampling rate of sensors at 1/500 s.

In this study, simple and easy-to-use sinusoidal motions were applied with frequencies ranging from 2 to 35 Hz and amplitudes of 0.05 to 1.4 g. A sinusoidal wave is characterized by a constant number of 50 cycles for all frequencies and amplitudes (see Supplementary Fig. S4a online). Employing a sine wave with a single frequency in each run allows for a comprehensive structural response analysis, revealing how stiffness and natural frequencies change in response to damage. Additionally, the obtained results can be utilized for the development of future numerical simulations. Sweep runs with an amplitude of 0.05 g and frequencies ranging from 2 to 50 Hz were performed to determine the natural frequency of the house model. The loading sequence followed a pattern from higher frequency to lower frequency and from lower amplitude to higher amplitude (see Supplementary Fig. S4b online). The loading sequence was arranged according to the Japan Meteorological Agency (JMA) intensity scale, which evaluates ground shaking experienced during an earthquake at specific locations^43^, as applied in some experiments^14,24^.

## Results

Supplementary Fig. S5 provides a summary of the experiment, including the number of runs and the level of damage. The evaluation of the damage level in the house model was assessed in accordance with the European macro-seismic scale 1998^44^. In this scale, D1 represents light structural damage, D2 indicates moderate structural damage, D3 denotes heavy structural damage, D4 represents partial collapse, and D5 indicates complete collapse. Run numbers 1 and 2, with amplitude 0.05 g and covering a frequency range from 2 to 50 Hz, were applied as a sweep run to determine the natural frequency and stiffness of the house models at an initial stage. The non-retrofitted house model had a natural frequency of 21.36 Hz, whereas the retrofitted house model showed slightly higher frequency of 25.63 Hz. Additionally, the initial stiffness measures 2.28 kN/mm for the non-retrofitted house model and 3.28 kN/mm for the retrofitted house model. The non-retrofitted house model experienced complete collapse at run 43 (0.8 g and 10 Hz), whereas the retrofitted house model exhibited complete collapse at run 56. Run 54 represents the maximum loading capacity on the shaking table, and both runs 55 and 56 shared the same amplitude and frequency as run 54 (1.2 g and 2 Hz).

### Lateral drift and hysteresis curves

**Figure 2:**
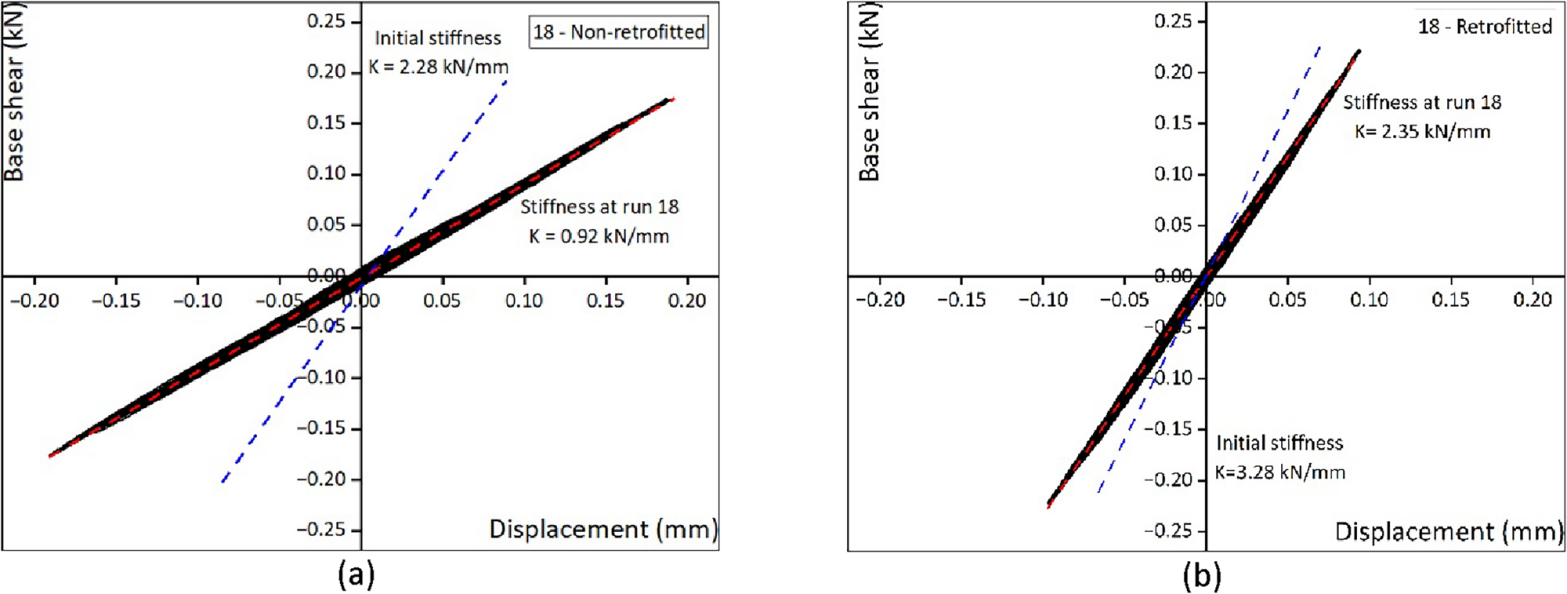
(**a**) Non-retrofitted at run 18 (0.1 g and 2 Hz), (**b**) retrofitted at run 18 (0.1 g and 2 Hz).

The behavior of the non-retrofitted and retrofitted house models was evaluated by plotting the measured displacement at the top (L4) (Fig. 1a) against the base shear. Figure 2 shows the hysteresis curve of non-retrofitted and retrofitted masonry house models, providing a visual representation of the force, displacement, and stiffness degradation. Figure 2 shows hysteresis curve of non-retrofitted and retrofitted house models, with the blue dashed line represents the initial stiffness of the house model obtained from the sweep test, whereas the red dashed line indicates the stiffness after run 18.This run was selected to compare the performance of both models in low frequency and amplitude. The non-retrofitted house model showed a peak displacement of ± 0.19 mm, which was higher compared to the retrofitted house model of ± 0.1 mm. Both models experienced a reduction in stiffness, where the non-retrofitted house model decreased from 2.28 kN/mm to 0.92 kN/mm, and the retrofitted house model decreased from 3.28 to 2.35 kN/mm. The reduction in stiffness indicates that the house model experienced cracking or damage on the masonry wall. However, in the retrofitted house model, the cracks were not visible as they were covered by the FR-Paint. The stiffness continued to decrease until the structure completely collapsed, the hysteresis curve for other runs is presented in Supplementary Figs. S6–S8.

### Cracks pattern & failure mechanism

#### Non-retrofitted masonry house

Since the shaking was unidirectional from north to south, the east and west walls were subjected to in-plane loading, while the north and south walls were subjected to out-of-plane load direction. After the construction, the house models were carefully transported to the shaking table using a crane, ensuring a slow and smooth movement to prevent any cracks or failure during transport. Nevertheless, due to the weak characteristics of URM, hairline cracks emerged, particularly in the gable walls. Until run 17 (at 0.1 g and 5 Hz), the structure did not exhibit any significant damage. However, following run 18 (0.1 g and 2 Hz), long-horizontal hairline cracks were observed in the gable walls (see Supplementary Fig. S9 online). After run 25, the gable walls on north the north and south walls collapsed simultaneously. The non-retrofitted house model exhibited a cracking behavior similar to that observed in the full-scale experiment, highlighting the gable wall as the most vulnerable part in the non-retrofitted house model^5^. This vulnerability comes from a lack of lateral support to adjacent walls, the absence of reinforcement, and the gable wall exhibiting large displacement compared to other parts. In the retrofitted house model, a preventive measure was taken by applying FR-Paint to connect the roof purlin with the gable top to enhance gable’s resistance to lateral forces.

**Figure 3:**
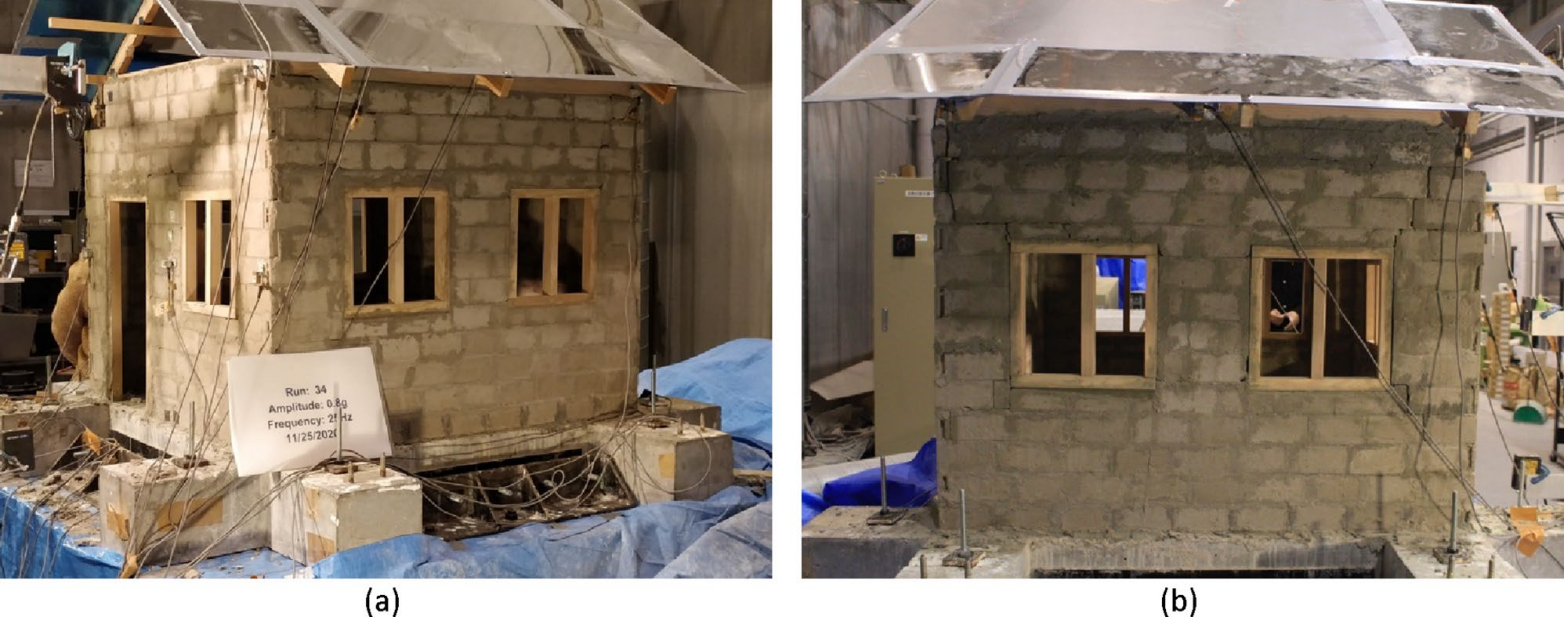
Cracks pattern of the non-retrofitted house model after run 34. (**a**) Out-of-plane failure on the north and south walls, (**b**) in-plane failure on the east and west walls.

Figure 3a shows the house model after run 34, the non-retrofitted house model sustained severe damage, with some blocks falling, particularly those located in the top left of the south wall. Vertical splitting cracks were observed in the north and south walls in an out-of-plane direction. This type of failure was not seen in the retrofitted house model. Figure 3b shows the east and west walls, subjected to in-plane loading, experienced significant cracking around openings. The presence of openings, such as windows, changes the stress distribution within the section, resulting in increased stress concentrations^45^. This localized increase in shear stress has the potential to initiate diagonal cracks from the corners of windows. Cracks expanded and propagated from openings to the corners (either right-top or left-top). Following run 41 (0.4 g and 10 Hz), the non-retrofitted house model sustained significant damage with the structure partially collapsing. In the subsequent runs, run 42 and 43, the amplitude was increased while adhering to the same frequency of 10 Hz. After run 42 (0.6 g and 10 Hz), the house model house was severely damaged, the cracks became more extensive, and some blocks fell (see Supplementary Fig. S10 online). Diagonal x-type shear cracks were observed on the east and west walls. Cracks also appeared in the masonry unit, as expected, owing to the low-strength characteristics of CHB. The openings were almost separated before the structures completely collapsed (see Supplementary Fig. S11 online). The non-retrofitted house model collapsed at the beginning of run 43 (0.8 g and 10 Hz), corresponding to a JMA intensity of approximately 4.

**Figure 4:**
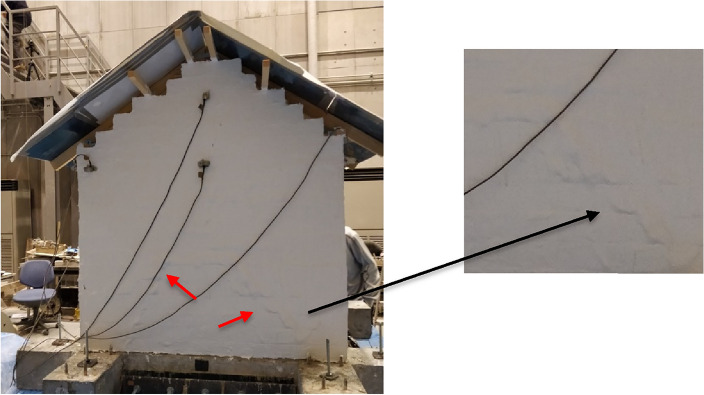
Cracking pattern of the retrofitted house model. The pictures were taken following the 48th run, and bubbles were forming on the surface of the masonry. The pattern resembles diagonal bubbles, extending from the bottom corners to the top of the masonry walls. Bubbles emerged on both the south and north walls, subjected to out-of-plane loading. Until this run, there were no indications of damage on the east and west walls in the in-plane direction.

Figure 4 shows the crack patterns and failure mechanism of the retrofitted house model subjected to various input motions. The retrofitted house model did not show any significant damage until run 36 (0.6 g and 20 Hz). The first sign of damage was observed at run 37 (0.8 g and 20 Hz), bubbles began to emerge on the wall surface due to cracks on the masonry surface and the delamination of the paint layer. After run 41 (0.4 g and 10 Hz), the bubbles were developing on the outside and inside of the masonry house. These bubbles enlarged and extended from the gable wall to the bottommost wall, eventually transforming into a rip in the subsequent runs (see Supplementary Fig. S12 online). Supplementary Fig. S13 shows a rip that appeared near the openings in the north wall after run 48 (1 g and 5 Hz), with no significant damage observed on the west and east walls. The gable wall was tied to the roof structure using FR-paint, and despite the partial detachment of the lowermost layer of the north and south walls from the base, the overall structures remained standing. After run 53 (0.8 g and 2 Hz), the south wall collapsed, but the whole house model remained standing even after some additional runs (see Supplementary Fig. S14 online). Even after run 55 (1.2 g and 2 Hz), there was no damage observed to the west and east walls. The retrofitted house model finally collapsed after run 56 (1.2 g and 2 Hz) at an intensity JMA of 7.

### Performance-based on Arias intensity

**Figure 5:**
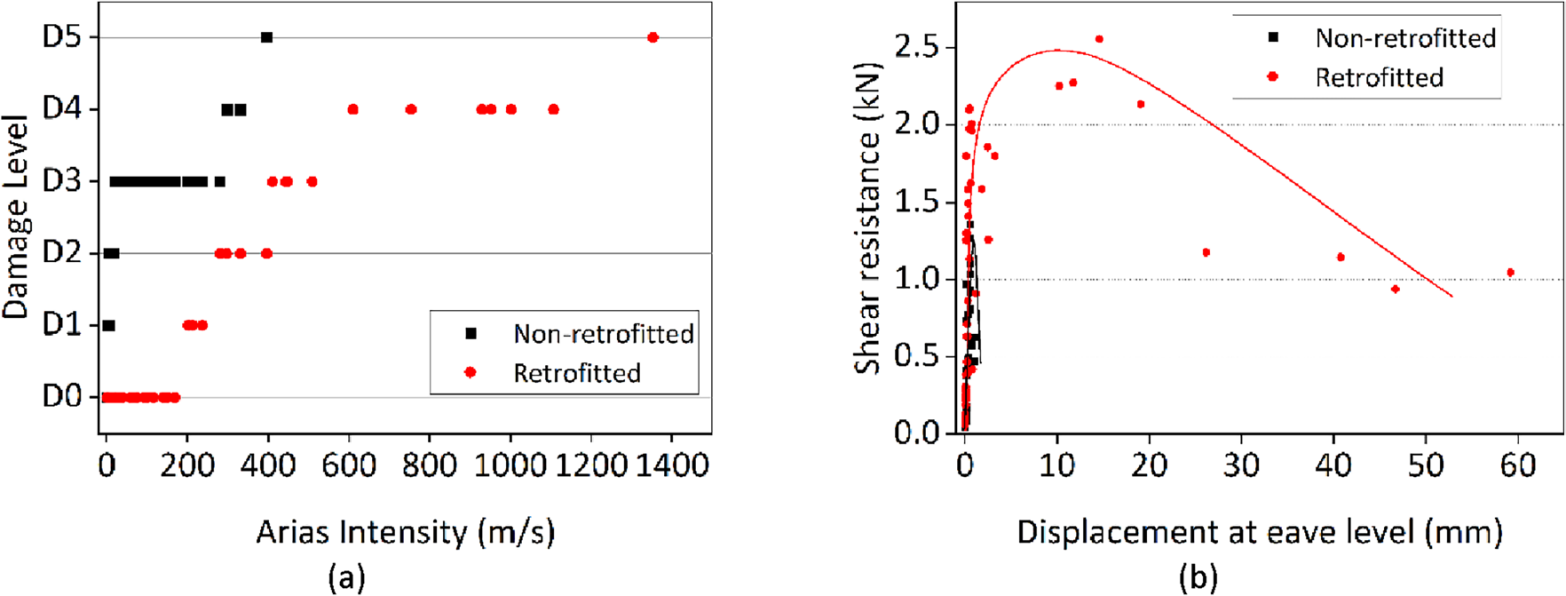
(**a**) Arias Intensity corresponds to damage levels D1-D5 based on the European macro-seismic scale, (**b**) shear resistance capacity.

The Arias intensity (${I}_{a}$) measures the accumulated energy transferred to the structure through the ground motion during seismic events. It can serve as a measure of shaking intensity by integrating the cumulative ground motion intensity from the acceleration record at the base over the total shaking duration, which has been adopted in some studies^14,35^. This measure assesses the total seismic energy absorbed by the ground^46^. Figure 5a shows the performance levels of each specimen against various input motions. From the results, the retrofitted house model has the highest Arias intensity compared to non-retrofitted house model.

## Discussions

### Lateral deformation

**Figure 6:**
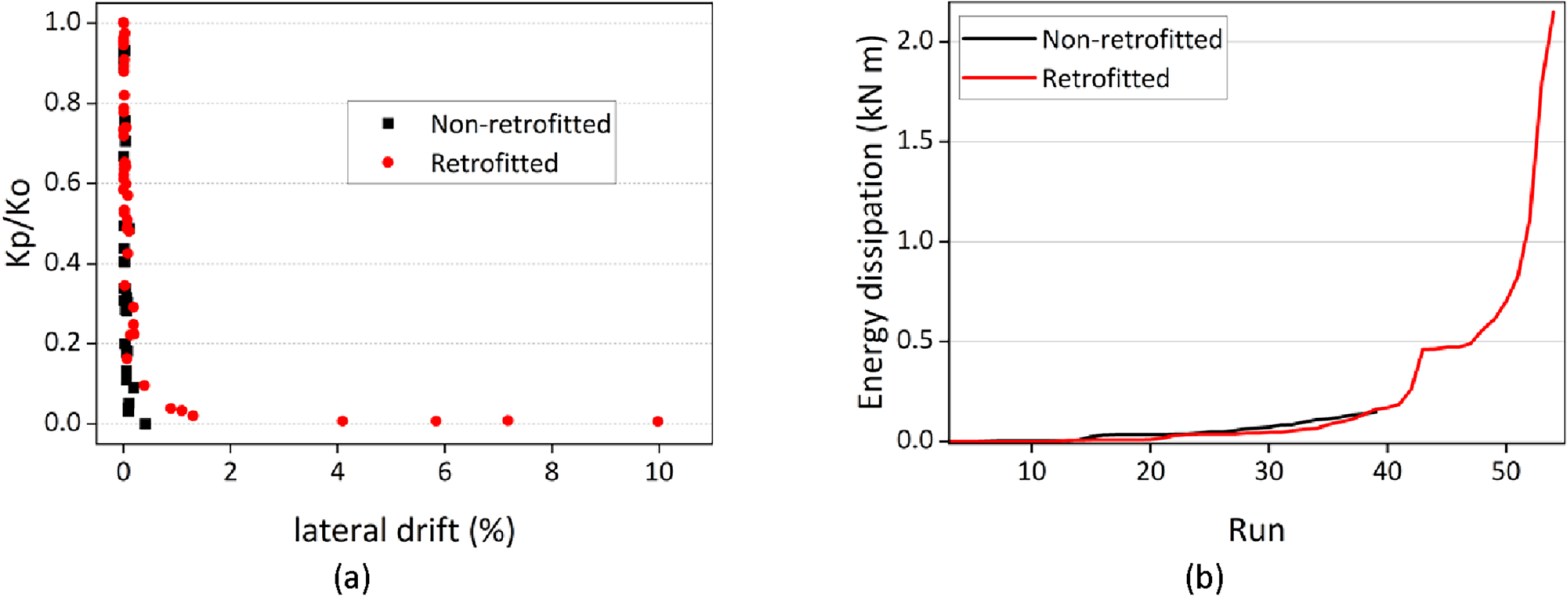
(**a**) Variations in the stiffness of the test models, (**b**) Cumulative energy dissipation of the house models.

The retrofitted house model outperformed non-retrofitted house model, demonstrating a higher displacement, as illustrated in Fig. 6a, which depicts a drift ratio of 9.97% for the retrofitted house model. At run 25, the non-retrofitted house model suffered heavy structural, marked by the collapse of gable walls on north and south sides, occurred at a drift ratio of approximately 0.193%. Some accelerometers from non-retrofitted house model were removed after run 37, and the highest recorded drift ratio was 0.4% at run 40. In contrast, the retrofitted house model showed structural integrity without exhibiting any signs of damage until run 36. The gable wall of the retrofitted house model was still attached to the structure until the entire wall collapsed. These results suggest a potential improvement of at least 18 times in the deformation capacity of reduced-scale masonry houses when utilizing FR-Paint. Therefore, it is imperative to highlight the necessity for future investigations using full-scale house model to investigate the effectiveness of FR-Paint.

### Stiffness degradation

From Fig. 6a, the stiffness was related to the degree of damage to the masonry house model. The stiffness continued to decrease toward the end of the run as the masonry structures experienced more damage. The effective stiffness was defined as the slope of the hysteresis curve. The stiffness degradation of a masonry wall was evaluated by comparing the initial stiffness and final stiffness for each run. The initial stiffness (Ko) was calculated at the sweep run for both house models, and the final stiffness (Kp) was calculated for representative cycles in each run. Although the results of both sweep tests were almost the same, sweep test run number two was selected as it was applied before the actual input motion. Figure 2 and Supplementary Figs. S6–S8 show the hysteresis curve along with its corresponding slope for runs 18, 23, 37, 42, and 48. From Fig. 6a, reduced stiffness was observed even at a low drift ratio. Even in a small displacement shaking, the non-retrofitted house model experienced a displacement that was not restored to its original position. Despite the observation of the first sign of damage at run 37 in the retrofitted house model, the stiffness was already in decline, indicating that the masonry units within the paint had broken, and there was a loss of mortar bonds.

### Shear resistance capacity

Figure 5b shows the relationship between shear resistance against maximum displacement at the eave level for both non-retrofitted and retrofitted house models. Shear resistance capacity involves evaluating lateral shear force acting on masonry house models during dynamic loading by comparing the maximum shear in the house model with the corresponding maximum eave displacement for each run. In small displacements, both non-retrofitted and retrofitted house models exhibited comparable lateral shear forces. However, as significant cracks developed to the non-retrofitted house model, the shear resistance decreased, contrasting with the retrofitted house model that maintained a higher shear resistance. This observation highlights the impact of FR-Paint on the lateral resistance performance of the masonry structures. The lateral shear capacity of the retrofitted house model began to decrease at displacement of ± 15 mm, due to significant cracks and partial detachment of the bottom layer of the masonry wall from the foundation.

### Energy dissipation

A comparison of results of energy dissipation capacities between the non-retrofitted and retrofitted house models for the corresponding run are shown in Fig. 6b. The energy dissipation capacity was evaluated by calculating the area enclosed within the hysteresis loops formed by the measure base shear and displacement at the top (L4). From Fig. 6b it is clear that the non-retrofitted house model had a very poor energy dissipation capacity, and heavy structural damage occurred to the model at low displacement. The retrofitted house model had a very high energy dissipation capacity. The dissipated hysteretic energy was mainly affected by the damage propagation mechanism. In the case of the retrofitted house model, the use of FR-Paint enhanced the integrity of masonry elements and prevented the house model from experiencing a sudden drop or brittle failure. In the non-retrofitted house model, the cracks propagated quickly after the first sign of the cracks. However, in the retrofitted house model, the crack propagated slowly. The energy dissipation capacity of the house model retrofitted with FR-Paint appeared to be at least 13 times larger than that of the non-retrofitted house model.

## Conclusions

Two masonry house models were subjected to shaking table testing to observe their dynamic behavior. The first house model depicted a typical residential house without reinforcement, while the second house model, retrofitted with fiber-reinforced paint, represented our proposed retrofitting approach. From the results of the shaking table testing experiment of retrofitted and non-retrofitted house models, the following conclusions were drawn:

1. The painting process using FR-Paint was simple, employing approximately 7 kg of FR paint, and the comprehensive painting of all interior and exterior walls was accomplished within 2 h. This study provides insights into the potential utilization of fiber-reinforced paint as an alternative retrofitting technique, demonstrating that FR-Paint can improve deformation capacity and energy dissipation. Additionally, it is important to highlight that specific preparatory steps are essential before applying FR-Paint, such as cleaning the masonry wall to ensure the effectiveness of the paint.
2. The initial sign of damage observed in the retrofitted house model is the appearance of bubbles, indicating cracks on the masonry surface and delamination of the paint layer. For future research, it is recommended to incorporate a primer before applying the paint to create a smooth and uniform masonry wall, enhancing paint adhesion strength^21^.
3. The main target of FR-Paint is to retrofit existing masonry; however, given the possibility of existing damage before FR-Paint application, future studies should determine the threshold of masonry damage at which FR-Paint maintains its effectiveness. Additionally, to counter moisture penetration from groundwater, additional measures should be explored. Although FR-Paint can prevent water penetration from the wall surface, there remains a potential for moisture to infiltrate the masonry wall from the ground. Once moisture infiltrates the masonry wall, it may be trapped due to limited evaporation, resulting in masonry deterioration and debonding of paint from the wall surface.
4. A scaled model experiment is understandable and can capture the general behavior of masonry houses, particularly when facing challenges such as equipment limitations, time constraints, and cost considerations. Nevertheless, in future research, it is imperative to conduct full-scale experiments employing construction materials representative of those found in developing countries, with adjustments to the fiber ratio and thickness of FR-Paint to achieve the desired strength.
5. The use of the sine-wave loading input can serve as a validation tool in numerical simulations. Future works should prioritize incorporating the use of recorded ground motion, as actual earthquakes exhibit a broad range of frequencies that would be necessary to observe in the behavior of the structure.

Shaking table testing of two masonry house models revealed the effectiveness of FR-Paint in improving deformation capacity and energy dissipation. The simplicity of application of FR-Paint to masonry walls presents a promising potential for widespread adoption in both developing and developed countries, aligning with common practices for painting. Recommendations include incorporating a primer for enhanced adhesion, determining damage thresholds for pre-existing conditions, and emphasizing real-scale experiments.

### Supplementary Information

Supplementary Figures.

Supplementary Table 1.

## Data Availability

The datasets used and/or analyzed during the current study are available from the corresponding author on reasonable request.
