# Supplementary material for: Shaking table tests of a one-quarter scale model of concrete hollow block masonry houses retrofitted with fiber-reinforced paint
Source: Sci Rep. 2024 Apr 5;14:8041. doi: 10.1038/s41598-024-58365-4 (PMC10997642; doi:10.1038/s41598-024-58365-4)
Supplement: Supplementary file 1 — Supplementary Figures. [file 41598_2024_58365_MOESM1_ESM.docx]

**Shaking table tests of a one-quarter scale model of concrete hollow block masonry houses retrofitted with fiber-reinforced paint**

Zamzam Multazam^1^*, Kenjiro Yamamoto^1^, Kishor Timsina^1^, Chaitanya Krishna Gadagamma^1^, Kimiro Meguro^1^

^1^ Institute Industrial of Science, The University of Tokyo, Tokyo, 153-8505, Japan

* Corresponding authors: Zamzam Multazam (z-multazam@outlook.com)

# Supplementary information

| 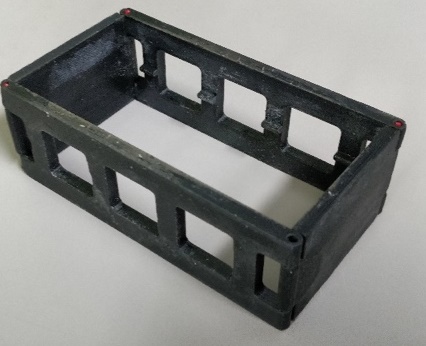  (a) | 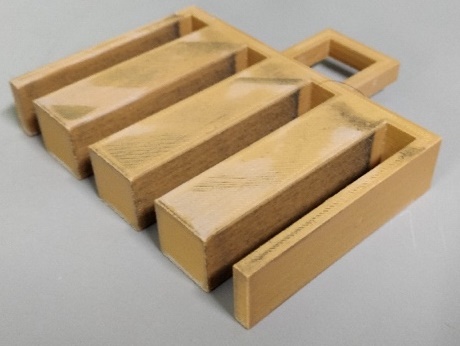  (b) | | 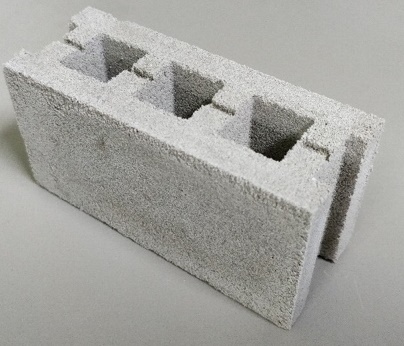  (c) |
| --- | --- | --- | --- |
| **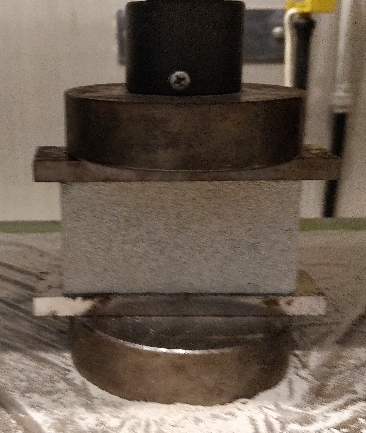**  (d) | | 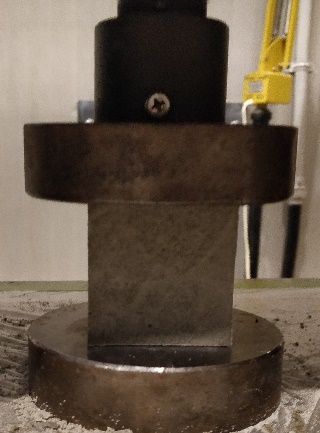  (e) | |

**Supplementary Figure S1.** CHB production and compressive strength testing of CHB and mortar. **(a)** CHB mold to create overall shape, **(b)** CHB mold to create holes in the center of blocks, **(c)** the quarter scale of CHB, **(d)** compressive strength testing of CHB, **(e)** compressive strength testing of cube mortar

| 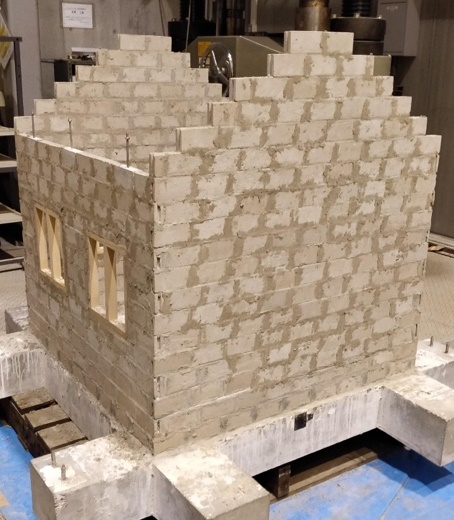  (a) | 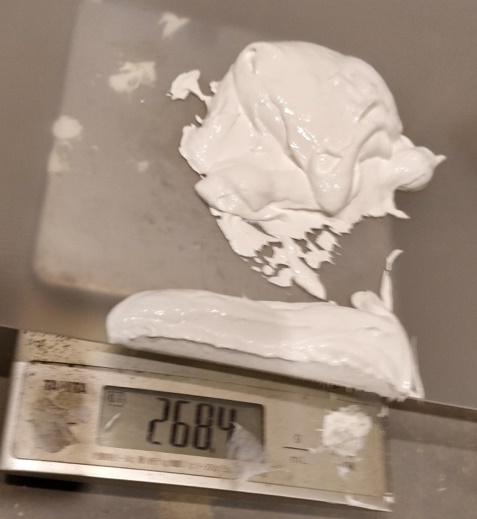  (b) |
| --- | --- |
| 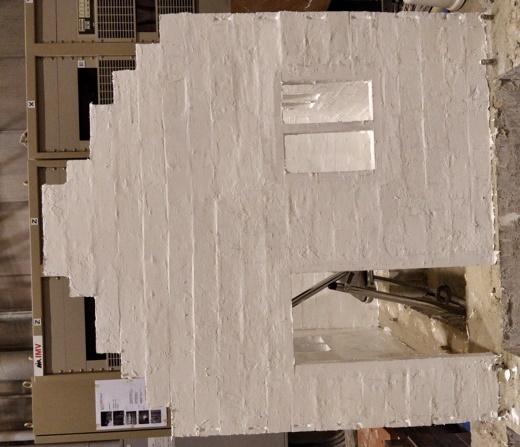  (c) | 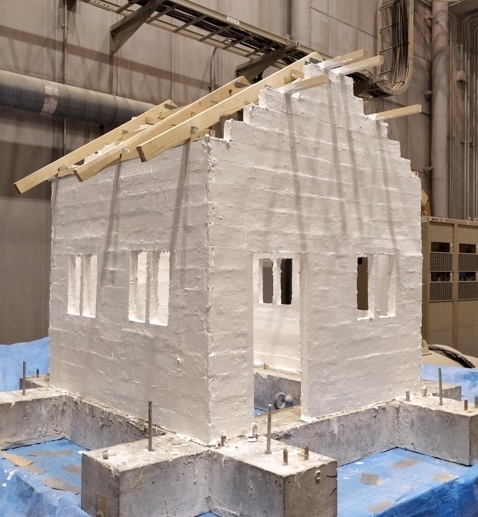  (d) |

**Supplementary Figure S2.** Retrofitting procedure of masonry structure**. (a)** Each model needed at least 500 CHBs, **(b)** the coating thickness was defined by measuring the weight of the paint, **(c)** the timber frame of the openings was painted, and purlin and masonry walls were also connected with paint, **(d)** house model after painting.


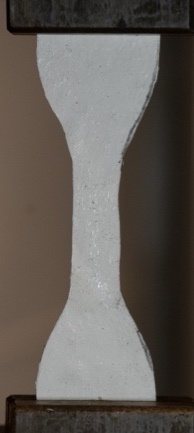


**Supplementary Figure S3.** Axial tensile testing of FR-Paint. The dumbbell shape of FR-Paint with a fiber ratio of 1% (by weight) was evaluated through an axial tensile test to estimate its tensile properties.

| 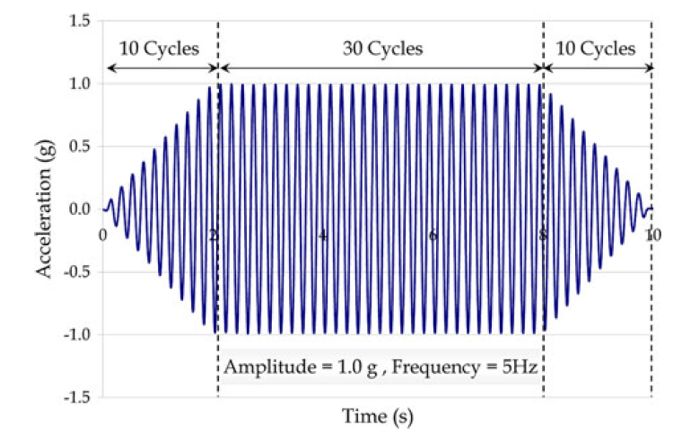  (a) | 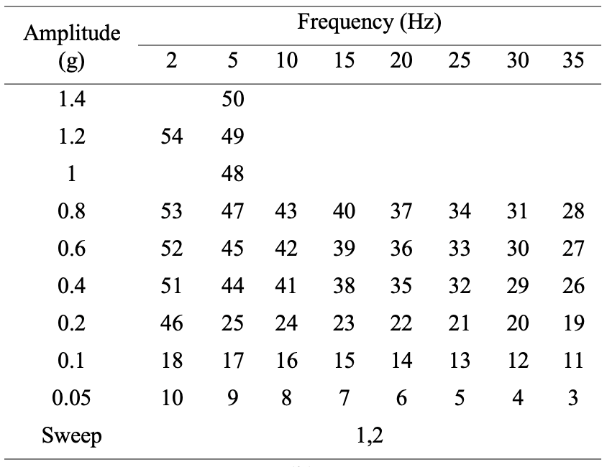  (b) |
| --- | --- |

**Supplementary Figure S4. (a)** Sinusoidal wave as the input motion, **(b)** the loading sequence for the shaking table test began with a progression from high to low frequency, followed by an increase in amplitude. The initial natural frequency of the non-retrofitted house model was found to be 21.36 Hz, aligning closely with the input motion frequency of 20 Hz. On the other hand, retrofitted house model exhibited a natural frequency of 25.63 Hz, corresponding closely to the input motion frequency of 25 Hz.

| JMA ~4 | JMA 5- | JMA 5+ | JMA 6- | JMA 6+ | JMA 7 |
| --- | --- | --- | --- | --- | --- |
|  |  |  |  |  |  |

| D0 | D1 | D2 | D3 | D4 | D5 |
| --- | --- | --- | --- | --- | --- |
| No damage | Light structural damage | Moderate structural damage | Heavy structural damage | Partially collapse | Complete collapse |

| \| Amplitude (g) \| Frequency (Hz) \| \| \| \| \| \| \| \| \| --- \| --- \| --- \| --- \| --- \| --- \| --- \| --- \| --- \| \| 2 \| 5 \| 10 \| 15 \| 20 \| 25 \| 30 \| 35 \| \| 1.4 \|  \|  \|  \|  \|  \|  \|  \|  \| \| 1.2 \|  \|  \|  \|  \|  \|  \|  \|  \| \| 1 \|  \|  \|  \|  \|  \|  \|  \|  \| \| 0.8 \|  \|  \| D5 \| D3 \| D3 \| D3 \| D3 \| D3 \| \| 0.6 \|  \|  \| D4 \| D3 \| D3 \| D3 \| D3 \| D3 \| \| 0.4 \|  \|  \| D4 \| D3 \| D3 \| D3 \| D3 \| D3 \| \| 0.2 \|  \| D3 \| D2 \| D2 \| D2 \| D2 \| D2 \| D2 \| \| 0.1 \| D2 \| D1 \| D1 \| D1 \| D1 \| D1 \| D1 \| D1 \| \| 0.05 \| D0 \| D0 \| D0 \| D0 \| D0 \| D0 \| D0 \| D0 \| | \| Amplitude (g) \| Frequency (Hz) \| \| \| \| \| \| \| \| \| --- \| --- \| --- \| --- \| --- \| --- \| --- \| --- \| --- \| \| 2 \| 5 \| 10 \| 15 \| 20 \| 25 \| 30 \| 35 \| \| 1.4 \|  \| D4 \|  \|  \|  \|  \|  \|  \| \| 1.2 \| D5 \| D4 \|  \|  \|  \|  \|  \|  \| \| 1 \|  \| D4 \|  \|  \|  \|  \|  \|  \| \| 0.8 \| D4 \| D3 \| D3 \| D2 \| D1 \| D0 \| D0 \| D0 \| \| 0.6 \| D4 \| D3 \| D2 \| D2 \| D1 \| D0 \| D0 \| D0 \| \| 0.4 \| D4 \| D3 \| D2 \| D1 \| D0 \| D0 \| D0 \| D0 \| \| 0.2 \| D3 \| D0 \| D0 \| D0 \| D0 \| D0 \| D0 \| D0 \| \| 0.1 \| D0 \| D0 \| D0 \| D0 \| D0 \| D0 \| D0 \| D0 \| \| 0.05 \| D0 \| D0 \| D0 \| D0 \| D0 \| D0 \| D0 \| D0 \| |
| --- | --- | --- | --- | --- | --- | --- | --- | --- | --- | --- | --- | --- | --- | --- | --- | --- | --- | --- | --- | --- | --- | --- | --- | --- | --- | --- | --- | --- | --- | --- | --- | --- | --- | --- | --- | --- | --- | --- | --- | --- | --- | --- | --- | --- | --- | --- | --- | --- | --- | --- | --- | --- | --- | --- | --- | --- | --- | --- | --- | --- | --- | --- | --- | --- | --- | --- | --- | --- | --- | --- | --- | --- | --- | --- | --- | --- | --- | --- | --- | --- | --- | --- | --- | --- | --- | --- | --- | --- | --- | --- | --- | --- | --- | --- | --- | --- | --- | --- | --- | --- | --- | --- | --- | --- | --- | --- | --- | --- | --- | --- | --- | --- | --- | --- | --- | --- | --- | --- | --- | --- | --- | --- | --- | --- | --- | --- | --- | --- | --- | --- | --- | --- | --- | --- | --- | --- | --- | --- | --- | --- | --- | --- | --- | --- | --- | --- | --- | --- | --- | --- | --- | --- | --- | --- | --- | --- | --- | --- | --- | --- | --- | --- | --- | --- | --- | --- | --- | --- | --- | --- | --- | --- | --- | --- | --- | --- | --- | --- | --- | --- | --- | --- | --- | --- | --- | --- | --- | --- | --- | --- | --- | --- | --- | --- | --- | --- | --- |
| (a) | (b) |

**Supplementary Figure S5.** A summary of the shaking table test on two masonry models. D1-D5 represent five distinct damage categories as per the European Macro-seismic Scale 1998. In this study, the JMA intensity scale was derived based on the observed damage level in the retrofitted house model, serving as a benchmark to evaluate the comparative impact of identical ground motion on the non-retrofitted model. **(a)** Non-retrofitted house model, **(b)** retrofitted house model.

| 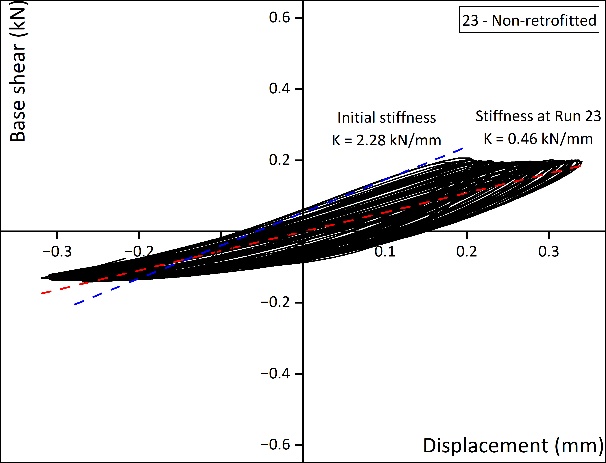  (a) | 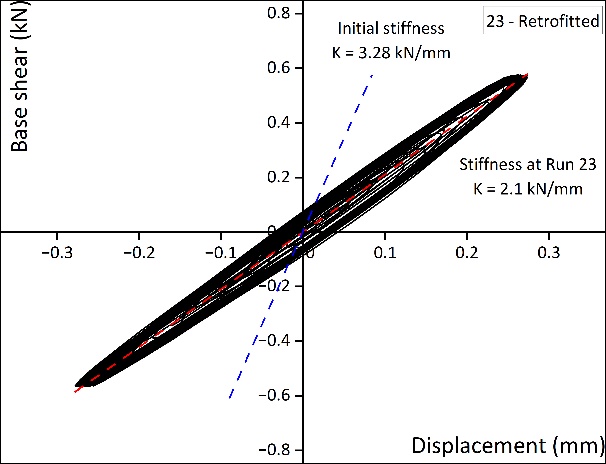  (b) |
| --- | --- |

**Supplementary Figure S6.** Hysteresis curve of the non-retrofitted and retrofitted model at run 23 (0.2 g and 15 Hz). **(a)** Run 23 was selected to observe both house models before the gable wall of the non-retrofitted model collapsed. The gable wall of the non-retrofitted model was heavily damaged after this run. The stiffness of the non-retrofitted model continued to decrease, showing a peak displacement of ±0.29 mm. From the hysteresis curve, the house model was experiencing cracks with higher displacement and was not restored to its original position. **(b)** The retrofitted house model also vibrated in the same displacement range as the non-retrofitted model. The retrofitted model with FR-Paint exhibited a better performance compared to the non-retrofitted model; No cracks were observed on the masonry surface, and the wall returned to its initial position with increased energy dissipation.

| 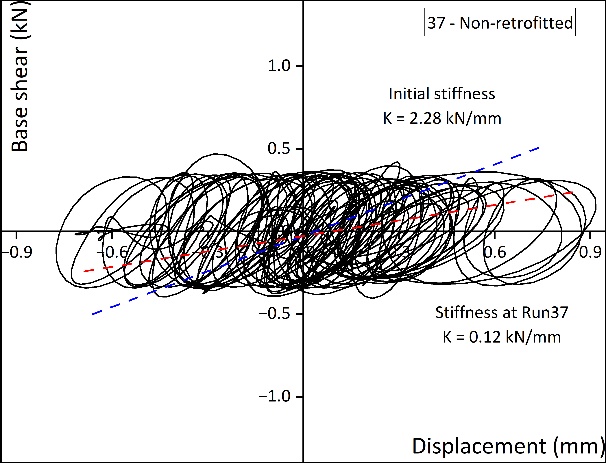  (a) | 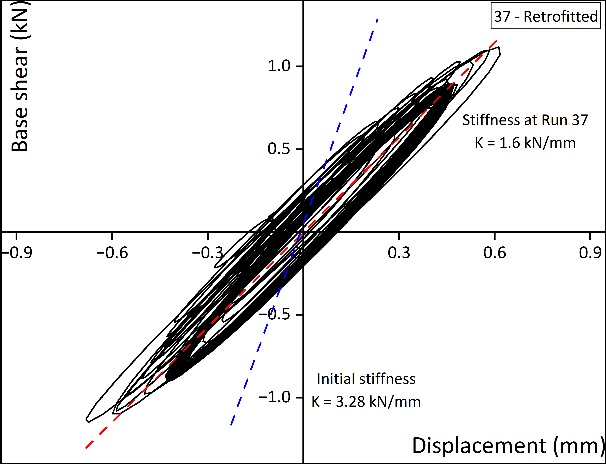  (b) |
| --- | --- |

**Supplementary Figure S7.** Hysteresis curve of the non-retrofitted and retrofitted model at run 37 (0.8 g and 20 Hz). **(a)** The non-retrofitted model had severe damage, especially around the openings. The masonry units almost fell and moved individually during shaking. The hysteresis curve showed that the masonry walls were moved and did not return to their original positions. After this run, some sensors on the non-retrofitted wall were removed to protect them from any possible damage. **(b)** Run 37 was selected to determine the behavior of both models after the appearance of the first sign of damage to the retrofitted model, marked by the appearance of bubbles on the masonry surface.

| 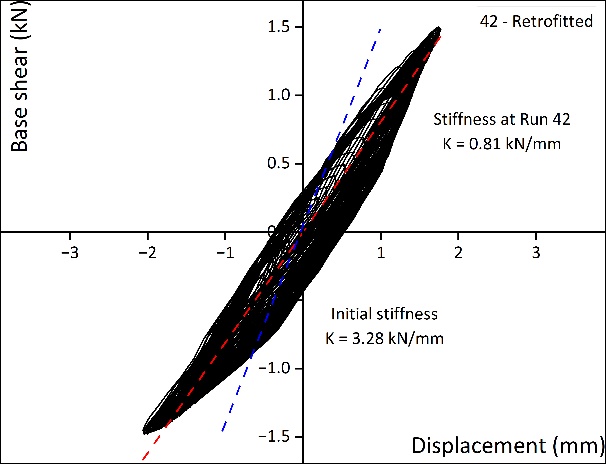  (a) | 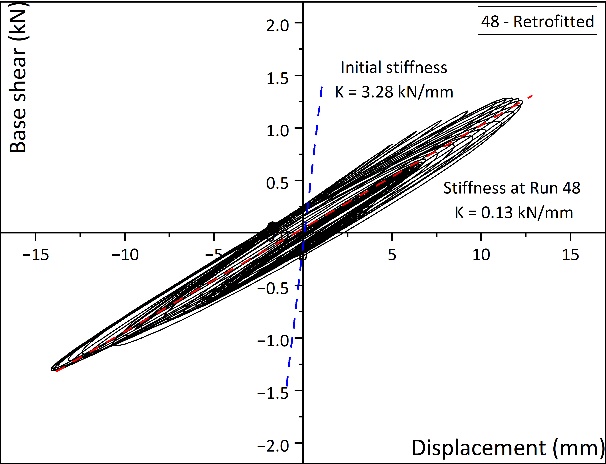  (b) |
| --- | --- |

**Supplementary Figure S8.** Hysteresis curve of the retrofitted model at run 43 (0.8 g and 10 Hz) and 48 (1 g and 5 Hz). **(a)** Even though the retrofitted house model exhibited a large displacement, the structure remained standing and could be shaken for more runs. Run 42 was the last run to record the response of the non-retrofitted house model since the structure was severely damaged. Non-retrofitted house model completely collapsed at run 43 (0.8 g and 10 Hz). **(b)** The retrofitted house model showed a peak displacement of ±9.24 mm at run 48. The hysteresis curve indicated that the retrofitted house model was forming cracks and was not restored to its original position. After this run, there was a partial detachment of the bottom layer of masonry from the base.

| 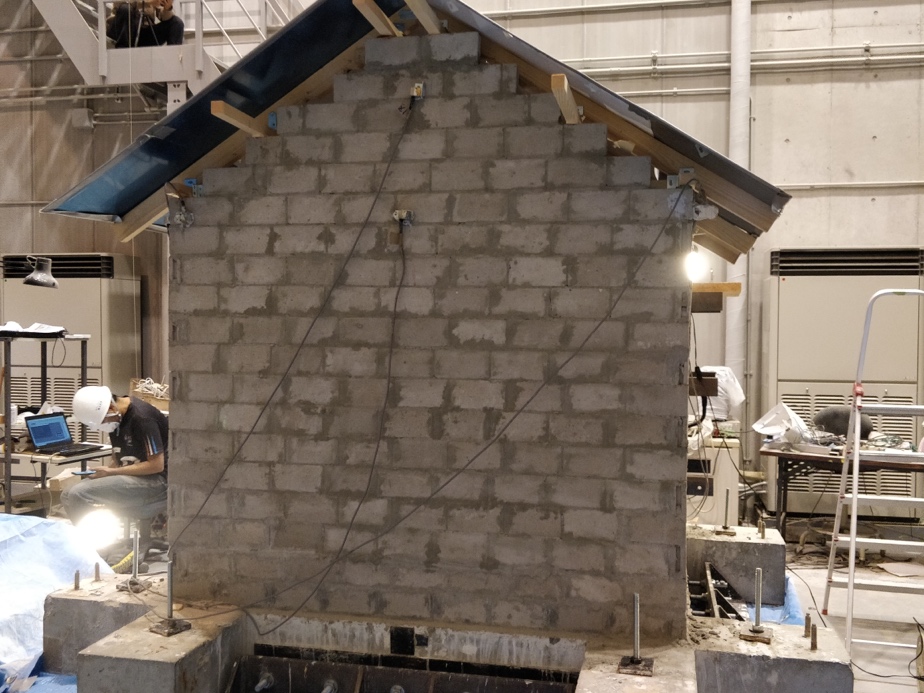 | 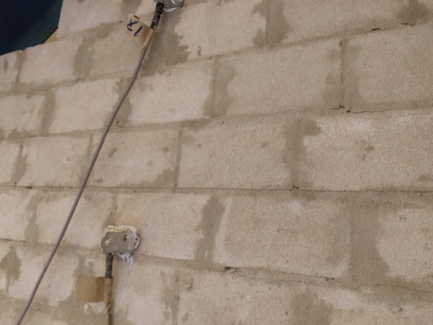 |
| --- | --- |

**Supplementary Figure S9.** Cracks pattern after run 18 (0.1 g and 2 Hz); hairline cracks along the façade (gable wall) became more apparent, and these cracks eventually led to the complete collapse of the gable wall in subsequent runs.

| 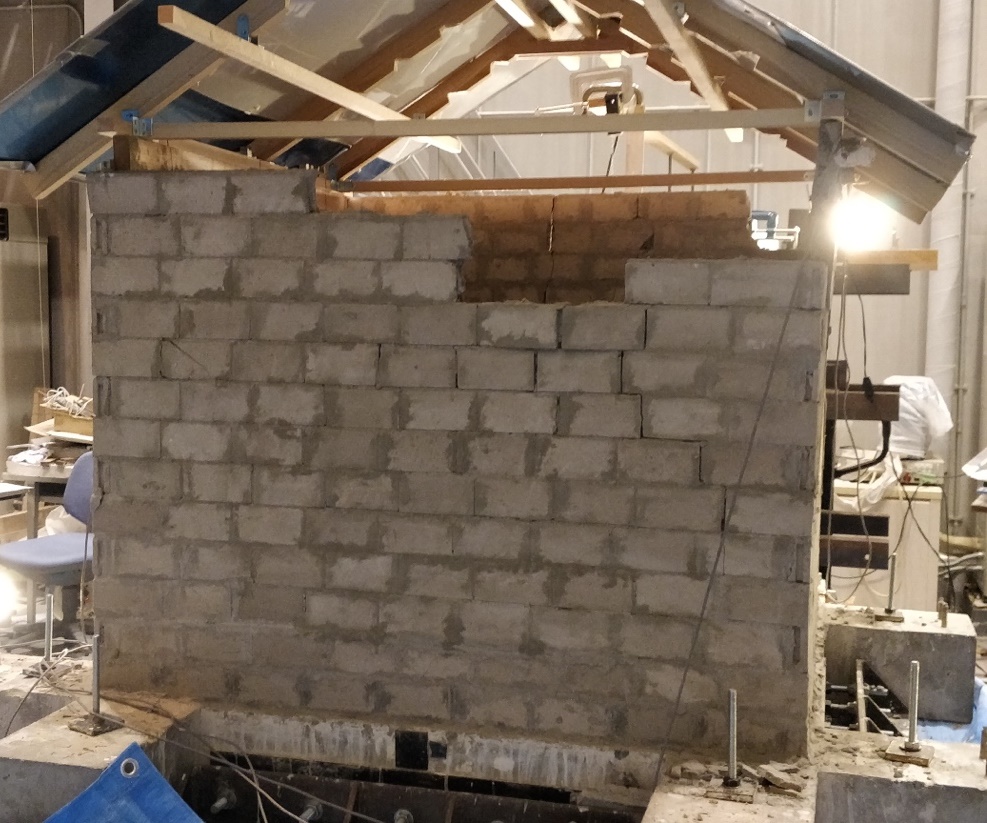  (a) |
| --- |
| 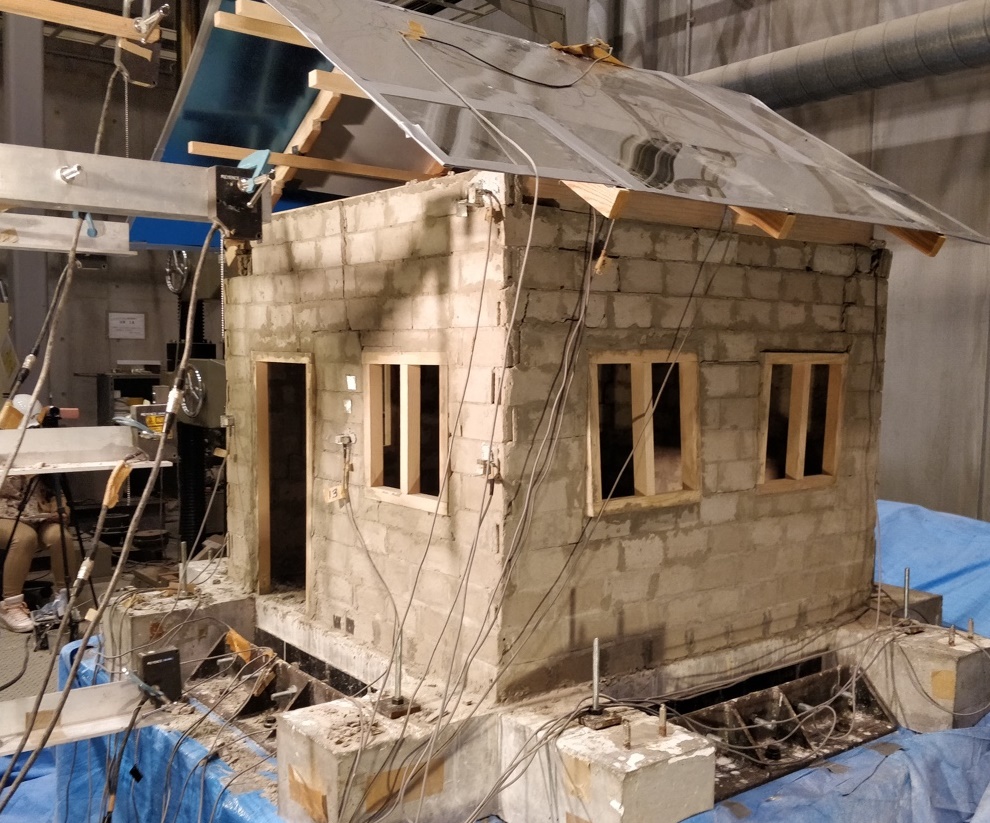  (b) |

**Supplementary Figure S10.** Cracks pattern of the south and north wall after run 42 (0.6 g and 10 Hz). This is the condition of the URM model just before complete collapse. The model was severely damaged with large cracks appearing on masonry surfaces. **(a)** In the north wall, Vertical cracks appeared in the corner and masonry ejection occurred (some blocks fell). **(b)** Many blocks from the top layers of masonry fell. During the shaking, most of the masonry elements were moving individually, indicating no connection between the masonry units.

| **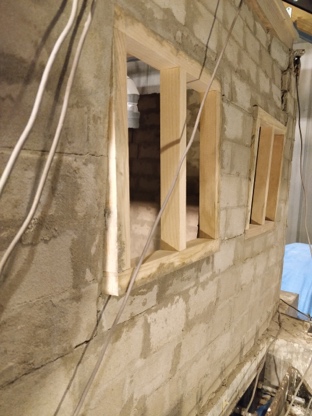** | **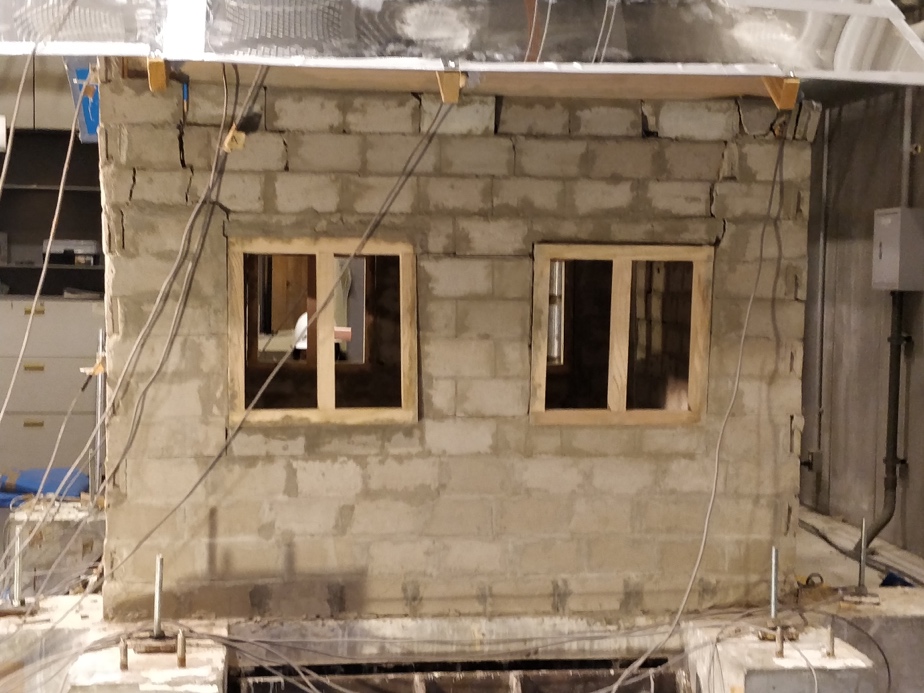** |
| --- | --- |

**Supplementary Figure S11.** Cracks pattern of the east and west wall after run 42 (0.6 g and 10 Hz). The windows on the east and west walls underwent significant displacement, almost separating from the wall. The complete failure at run 43 began with the windows completely separating from the wall.

| 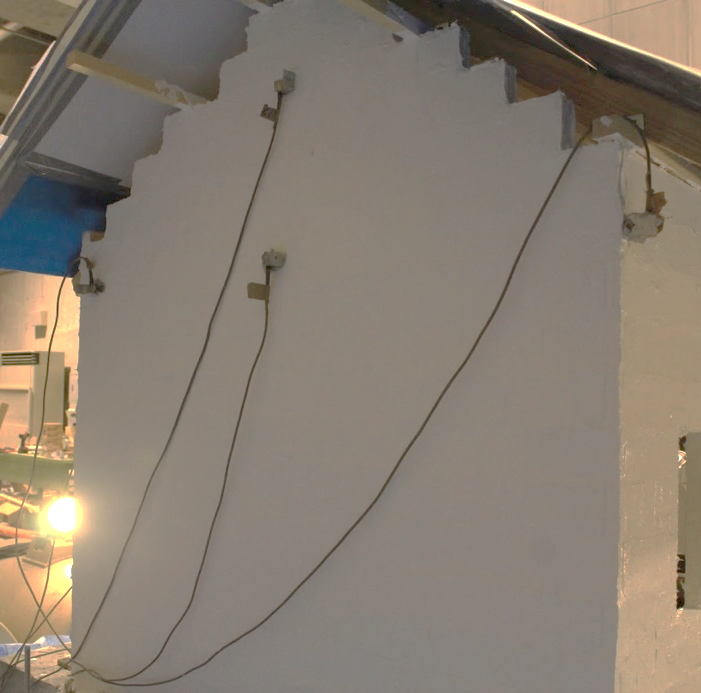 | 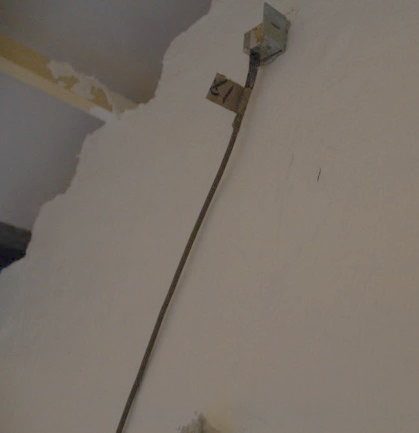 |
| --- | --- |

**Supplementary Figure S12.** Cracks pattern of retrofitted house model after run 41 (0.4 g and 10 Hz). The first bubble appeared on the gable wall after run 37 (0.8 g and 20 Hz). In the subsequent runs, the bubble enlarged, eventually transforming into a rip in run 41 (0.4 g and 10 Hz).

| 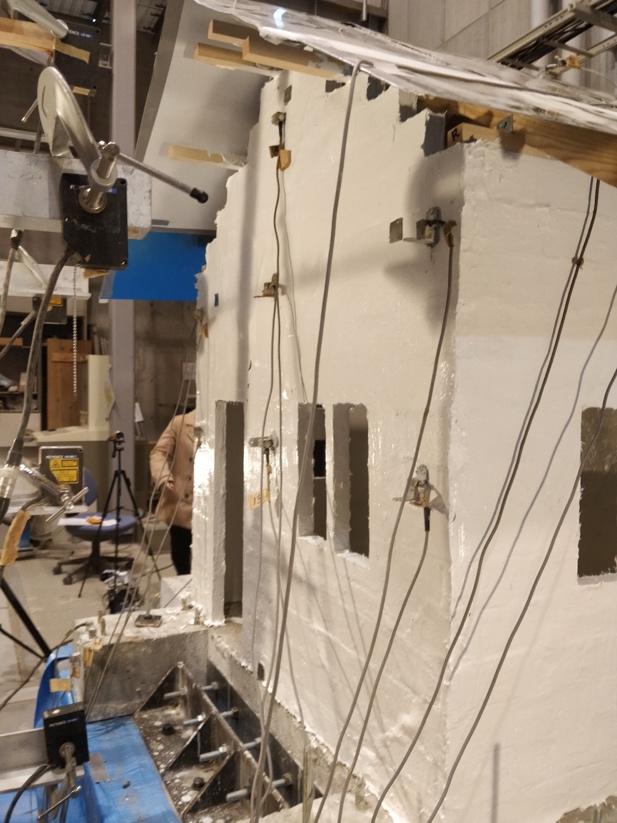 | 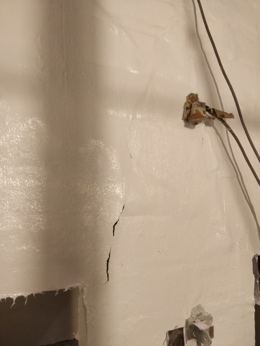 |
| --- | --- |

**Supplementary Figure S13.** Cracks pattern of retrofitted house model after run 48 (1 g and 5 Hz). The rip, indicated by the red dashed circle, continued to expand, and the bottom layer was detached from the base, as indicated by the red arrow.

| 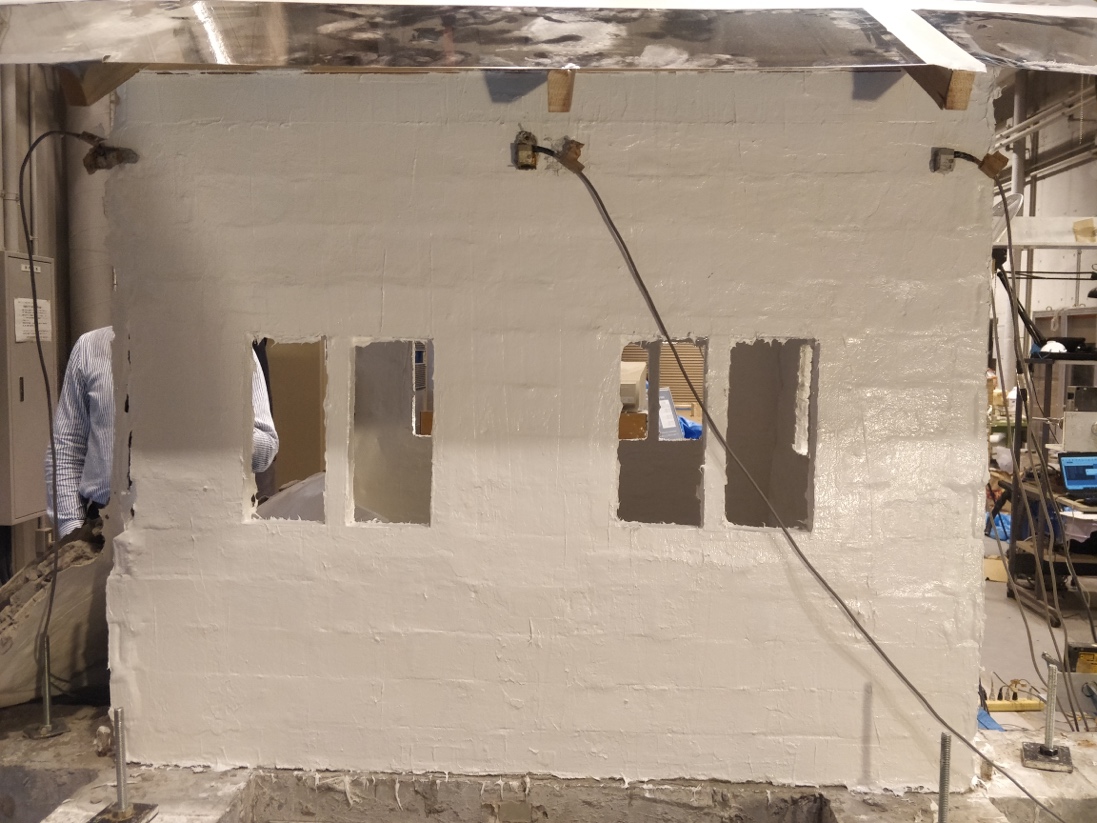  (a) |
| --- |
| 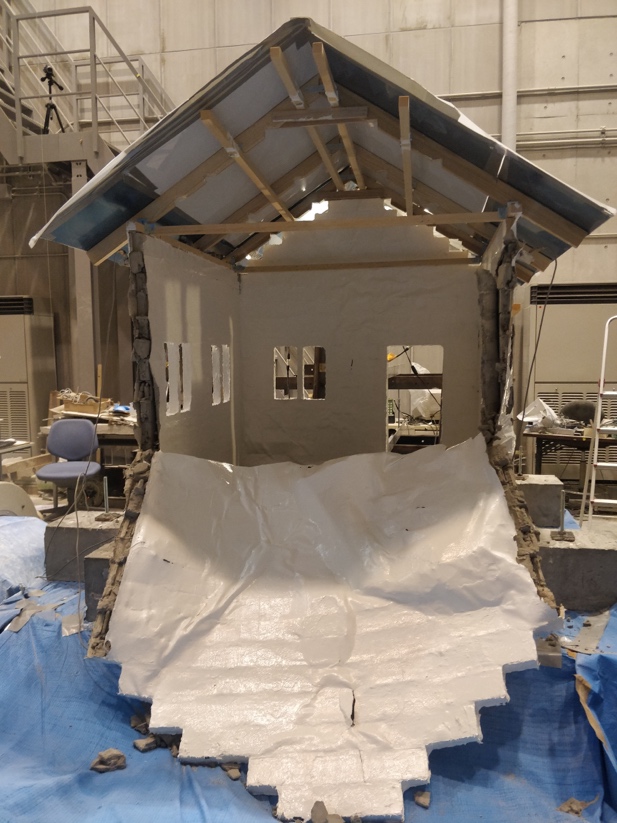  (b) |

**Supplementary Figure S14.** Cracks pattern of retrofitted house model after run 53 (0.8 g and 2 Hz). **(a)** No damages were observed in the east and west walls, **(b)** the south wall collapsed.
