# Supplementary material for: Shaking table tests of a one-quarter scale model of concrete hollow block masonry houses retrofitted with fiber-reinforced paint
Source: Sci Rep. 2024 Apr 5;14:8041. doi: 10.1038/s41598-024-58365-4 (PMC10997642; doi:10.1038/s41598-024-58365-4)
Supplement: Supplementary file 2 — Supplementary Table 1. [file 41598_2024_58365_MOESM2_ESM.docx]

# Table 1. Design Table

| Question | Hypothesis (if applicable) | Sampling plan (e.g. power analysis) | Analysis Plan | Interpretation given to different outcomes |
| --- | --- | --- | --- | --- |
| FR-Paint is a feasible and adoptable retrofitting solution in developing countries considering factors like material availability and cultural acceptability | FR-Paint, comprised of fiberglass, resin, and paint. These materials are available in developing countries. Its implementation is simple like ordinary paint, making it applicable to unskilled labor. | Compared with other retrofitting techniques found in the literature. | Evaluate and compare the simplicity of application, retrofitting procedures, time, and the overall effectiveness of each method. | Further investigation and improvement of the FR-Paint application might be necessary. |
| How can we address the low-strength characteristics of CHB to minimize masonry unit failure during the shaking, and what strategies are essential for preventing such failures? | The application of FR-Paint, providing full coverage to masonry walls, can effectively prevent the separation of individual blocks or their parts, leading to enhanced structural integrity. | The paint application should be uniformly painted across all masonry surfaces, and comparative analysis of failure patterns between the non-retrofitted and retrofitted house models. | Document the failure patterns for both models and generate visual representations (photos) to depict the distribution of these failure patterns. | Improving the adhesion strength between the retrofitting material and the masonry surface is essential to enhance the effectiveness of FR-Paint. |
| How well do scaled models represent the structural behavior of full-scale structures? | When appropriate geometrical and material scaling is applied, scaled models can replicate the seismic responses of full-scale constructions with some limitations. | Literature review on how previous scaled model experiments have been conducted. | Investigate and compare the crack pattern or failure behavior with other experiments featuring similar constructions. | Further experiments on full-scale models are necessary. |
| How effective is the FR-Paint in retrofitting one-story CHB masonry houses to improve the seismic capacity? | FR-Paint improves deformation and energy dissipation capacity of masonry house. | Two masonry models were constructed, represented non-retrofitted and retrofitted by FR-Paint. | Comparing its performance in terms of deformation capacity and energy dissipation | Reconsider the fiber ratio or coating thickness of FR-paint. To address unexpected results, suggestions for future work involve retrofitting procedures, and exploring additional countermeasures. |
| The shaking table will reveal the effectiveness of FR-paint as retrofitting material of masonry structures. | Retrofitted house model can withstand higher input motion and handle large displacement | Comparing two models, one non-retrofitted and the other retrofitted with FR-Paint, involves installing sensors during shaking to record data or capture images to analyze the failure behavior. | Examine failure modes, hysteresis curves, stiffness degradation, and the last run (when models experience complete collapse). | Examine the failure behavior and propose additional measures to address that failure. |
